# Understanding the Physicochemical Properties of Mitragynine, a Principal Alkaloid of *Mitragyna speciosa*, for Preclinical Evaluation

**DOI:** 10.3390/molecules20034915

**Published:** 2015-03-18

**Authors:** Surash Ramanathan, Suhanya Parthasarathy, Vikneswaran Murugaiyah, Enrico Magosso, Soo Choon Tan, Sharif Mahsufi Mansor

**Affiliations:** 1 Centre for Drug Research, Universiti Sains Malaysia, Penang 11800, Malaysia; 2 School of Pharmaceutical Sciences, Universiti Sains Malaysia, Penang 11800, Malaysia; 3 Advanced Medical & Dental Institute, Universiti Sains Malaysia, Bertam, Kepala Batas, Penang 13200, Malaysia; 4 Institute for Research in Molecular Medicine, Universiti Sains Malaysia, Penang 11800, Malaysia

**Keywords:** mitragynine, partition coefficient, pharmacology, pK_a_/pH, solubility, stability

## Abstract

Varied pharmacological responses have been reported for mitragynine in the literature, but no supportive scientific explanations have been given for this. These studies have been undertaken without a sufficient understanding of the physicochemical properties of mitragynine. In this work a UV spectrophotometer approach and HPLC-UV method were employed to ascertain the physicochemical properties of mitragynine. The pK_a_ of mitragynine measured by conventional UV (8.11 ± 0.11) was in agreement with the microplate reader determination (8.08 ± 0.04). Mitragynine is a lipophilic alkaloid, as indicated by a logP value of 1.73. Mitragynine had poor solubility in water and basic media, and conversely in acidic environments, but it is acid labile. In an *in vitro* dissolution the total drug release was higher for the simulated gastric fluid but was prolonged and incomplete for the simulated intestinal fluid. The hydrophobicity, poor water solubility, high variability of drug release in simulated biological fluids and acid degradable characteristics of mitragynine probably explain the large variability of its pharmacological responses reported in the literature. The determined physicochemical properties of mitragynine will provide a basis for developing a suitable formulation to further improve its solubility, stability and oral absorption for better assessment of this compound in preclinical studies.

## 1. Introduction

*Mitragyna speciosa* is an ethnomedicinal tree native to Malaysia, where it is named “Biak” or “Ketum”. It has traditionally been consumed as a leaves decoction for its stimulant effects to counter fatigue. Additionally, ketum has been employed, as a leaves poultice, to treat fever, diarrhea and for wound healing [1]. The other reported pharmacological properties of *M. speciosa*, include anesthetic, antinociceptive, analgesic and stimulant effects [2,3]. Recently, in the northern states of peninsular Malaysia that border Thailand, the consumption of ketum as a decoction has been popular [4]. Indeed, studies at these sites reported that ketum is widely sold as a decoction and regularly consumed by drug users to manage opiate withdrawal symptoms. However, a recent field study, suggests that ketum has abuse liability and can itself cause withdrawal symptoms following discontinuation after extended use [5]. Further studies in rodents suggest that mitragynine possesses potential threat of abuse beside its wide spectrum of therapeutic effects [6]. The usage of ketum is not limited only to the country of its origin. Over the past decade there has also been a growing interest in ketum usage outside this region as evidenced by the number of advertisements for its sale on the Internet [7]. 

**Figure 1:**
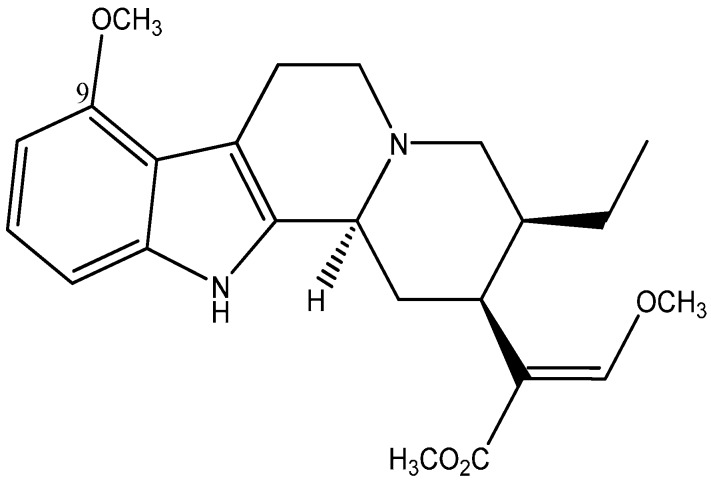
Chemical structure of mitragynine C_23_H_30_N_2_O_4_.

In general, the pharmacological effects of ketum are mainly attributed to its principal alkaloid mitragynine (Figure 1). Since then several pharmacological studies have been undertaken to evaluate this assertion objectively. However the mitragynine dose employed in these studies; analgesic (30–200 mg/kg) [8,9,10] pharmacokinetics (20–50 mg/kg) [11,12,13,14], toxicity (200–477 mg/kg) [9,10] varied largely across rodent species. Others reported no toxicity even at mitragynine dose levels of 800–900 mg/kg in rodents [15].

Most of these studies were carried out on empirical basis. For instance, the discrepancies observed in the pharmacokinetic properties have been attributed mainly to the analytical sensitivity of the mitragynine assays *per se* [12,14]. There could be other contributing factors such as chemical degradation, poor permeability across the gastrointestinal mucosa, pre-systemic elimination and first pass effect, but the poor aqueous solubility of mitragynine is evident. We find it is important to determine the physicochemical properties of mitragynine, even before one attempts to establish the metabolic properties or placing effort towards designing a more appropriate dosage form that mitigates the problem associated with mitragynine’s poor oral absorption. Hence, the physicochemical characterization of mitragynine is imperative. At present, mitragynine is at the preclinical stage and progressively gaining more attention as a potential substitute or adjunct drug therapy for addiction and pain [4,16]; hence the establishment of the physicochemical properties of mitragynine will be useful for development of a new medicinal drug. The present study is the first to determine the basic physicochemical properties of mitragynine such as pK_a_, solubility, log P and log D, *in vitro* drug release and stability in simulated body fluids and across selected pH values.

## 2. Results and Discussion 

### 2.1. Results

The wavelength of the maximum difference between ionized and neutral species of mitragynine in the corresponding UV spectrum was found to be at 248 nm. The pK_a_ of mitragynine was 8.11 ± 0.11 and 8.08 ± 0.04 by the conventional UV and microplate spectrophotometer methods, respectively (Table 1). 

**Table 1:** Typical mitragynine absorbance at various pH and determination of pK_a_ using the UV spectrophotometer and microplate spectrophotometer methods.

Target pH	UV Spectrophotometer Method			Microplate Spectrophotometer Method		
	Actual pH achieved	Absorbance (d)	pK_a_	Actual pH achieved	Absorbance (d)	pK_a_
7.6	7.52	0.8436	8.0219	7.55	0.4993	8.0860
7.8	7.78	0.8186	8.1617	7.76	0.4823	8.1124
8.0	7.96	0.7786	8.1726	7.94	0.4527	8.0256
8.2	8.13	0.7367	8.1810	8.15	0.4350	8.0870
8.4	8.34	0.6677	8.1281	8.32	0.4170	8.1015
8.6	8.45	0.6232	8.0489	8.50	0.3910	8.0257
8.8	8.71	0.6148	8.2692	8.66	0.3877	8.1477
9.0	8.92	0.5347	7.9303	8.80	0.3707	8.0567
Dm	0.4917 (pH 12)			0.3393 (pH 12)		
Di	0.9544 (pH 5)			0.5460 (pH 5)		
pK_a_ (Mean ± SD)	8.11 ± 0.11			8.08 ± 0.04		

Note: pK_a_ was calculated using the following formula: pK_a_ = pH + log (d − dm/di − d) for basic drug.

Analysis was performed on three different occasions to evaluate the repeatability of the techniques. The inter-day pK_a_ values obtained from different methods and with different concentrations are disclosed in Table 2. The solubility of mitragynine was found to increase from 18.7 ± 0.4 μg/mL in buffer at pH 9, to 64.6 ± 1.2 μg/mL in water and 88.9 ± 1.6 μg/mL in buffer pH 7, while being the highest at pH 4 buffer of 3.5 ± 0.01 mg/mL. For stability studies, the percentage recovered at 0.5 h, 6 h and 24 h of mitragynine in the buffer systems were 100.6 ± 0.3%, 91.8 ± 1.0% and 88.8 ± 1.4% for pH 4 buffer; 99.2 ± 0.4%, 96.1 ± 1.9% and 101.9 ± 0.9% for pH 7 buffer; 101.3 ± 2.6%, 97.8 ± 0.3% and 95.5 ± 2.5% for pH 9 buffer.

**Table 2:** Interday repeatability of the pK_a_ value.

Instrument	Day		
	1	2	3
UV Spectrophotometer	8.18 (0.10)	8.11 (0.05)	8.15 (0.12)
Microplate Spectrophotometer	8.18 (0.13)	8.10 (0.07)	8.12 (0.10)

As for HPLC validation study, the within day and day to day HPLC precisions and accuracies were found to be less than 5%. The calibration curve of mitragynine was linear over the concentration range of 0.16–20 μg/mL (r^2^ > 0.999). The lower limits of detection and quantification were 0.1 μg/mL and 0.16 μg/mL, respectively. This method was used for quantification of mitragynine in partition coefficient study. The n-octanol/water partition coefficients (logP and logD) obtained under various conditions are detailed in Table 3. Mitragynine has logP and logD (pH 4) values of 1.70 and 0.78, respectively.

**Table 3:** The *n*-octanol-water partition coefficient of mitragynine.

Solvent Layer	Mitragynine Content (µg/mL) at Different Buffer pH			
	pH 4 **	pH 7 **	pH 9 **	Water *
Octanol	17.97	20.54	18.45	20.74
Buffer	2.98	0.38	0.51	0.42
Calculated *n*-octanol/water partition coefficient	0.78	1.73	1.56	1.70

Note: *: logP; **: logD.

Table 4 shows the results of pure mitragynine stability after incubation in SGF and SIF respectively. In SGF study, from the 20 min time point onwards, the relative deviation (%) of mitragynine was greater than 20%.

**Table 4:** Mitragynine stability in SGF and SIF (with enzymes).

Time (min)	SGF	RD (%)	SIF	
	Concentration Found (µg/mL)		Concentration Found (µg/mL)	RD (%)
0	18.72 ± 0.05	-	6.70 ± 0.60	-
10	18.68 ± 0.23	−0.21	6.69 ± 0.63	−0.07
20	14.98 ± 0.88	−20.00	6.56 ± 0.67	2.06
30	14.54 ± 0.58	−22.5	6.99 ± 0.21	4.43
40	14.19 ± 0.37	−22.31	7.03 ± 0.13	4.94
50	14.11 ± 0.22	−24.19	6.93 ± 0.13	3.49
60	13.94 ± 0.20	−25.53	6.86 ± 0.01	2.49
120	-	-	6.78 ± 0.79	1.31
180	-	-	6.93 ± 0.77	3.46

**Figure 2:**
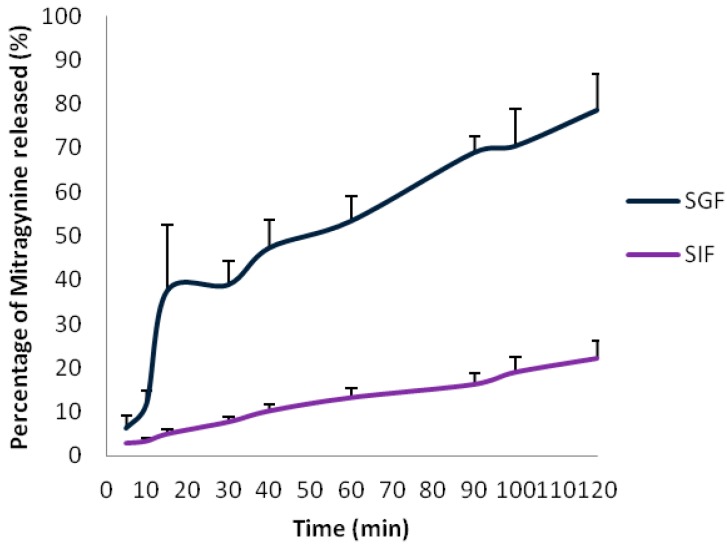
Dissolution profiles of mitragynine in SIF and SGF (*n* = 3).

In contrast, for the SIF study throughout the incubation period the relative deviations (%) of mitragynine was found to be no greater than ±5%.The drug dissolution profiles over time for capsule filled with mitragynine standard in SIF and SGF are shown in Figure 2. In SGF, mitragynine dissolution was inconsistent at early dissolution time points and only 80% of the drug was released up to 2 h. The RSD of >50% was observed at early dissolution time points (5 to 15 min) but for the rest of the period the RSD was <20%. As for SIF, the drug release was very slow and incomplete; as only 22% of the drug was released up to 2 h. A RSD of >20% was noted throughout the dissolution period.

### 2.2. Discussion

Poorly water soluble alkaloids, such as mitragynine, require a suitable strength of stock solution for pK_a_ determination. Mitragynine stock solutions in the range 5–40 μg/mL were tested in various buffers. In agreement with values suggested for poor solubility compounds [17] the optimal testing concentration was found to be 50 μM (20 μg/mL) for conventional UV and 12.5 μM (5 μg/mL) for microplate method. The present study is the first to determine the pK_a_ value for mitragynine which is in agreement with an *in silico* predicted value of 8.3 [18]. The pK_a_ values determined by conventional and microplate spectrophotometer methods were comparable and showed good assay repeatability (Table 2). There are several advantages for the microplate spectrophotometer method over the conventional assay. The former is rapid and cost effective, and requires only a small volume of sample and a low drug concentration for spectrum analysis, hence making the method suitable for analysis of mitragynine.

We found that acetic acid, Tris base and sodium carbonate buffer systems provided an improved buffering capacity over phosphate buffers which are commonly used in the range of pH 3 to 12 [19,20]. In addition, these buffers did not absorb UV at the selected analytical wavelength, thus no interference was encountered in the spectrophotometer assay. In both assay procedures, similar buffer solutions were used for UV spectrum analysis. The UV absorbance spectra of various absorbing species at different pH showed a gradual decrease in absorbance beyond pH 7.0 for both methods. The mitragynine absorption spectrum was higher in the acidic pH when compared to the basic media. Mitragynine is a weak basic compound and it exhibits a basic character that is attributed to an amine group. This is supported by the fact that it is highly soluble at acidic pH, but the solubility was extremely reduced in basic media. Mitragynine exists predominantly in an ionized form at lower pH; hence a higher solubility was seen at lower pH. In the present study, mitragynine lipophilicity was determined by the octanol/water) partition method. Lipophilicity is expressed in several different ways including terms such as logP and logD. LogP is calculated as a ratio of concentrations of unionised compound between the octanol and water. LogD reflects the distribution of both neutral and ionized species of a drug at a particular pH between the aqueous and organic phase, and therefore is pH dependent. Mitragynine has an intermediate lipophilicity, with a logP value of 1.70. Highly lipophilic compounds are more exposed to P_450_ metabolism and associated with increase in non-specific binding to plasma proteins that limit blood brain barrier (BBB) penetration [21]. Those drugs with intermediate lipophilicity often exhibit highest *in vivo* brain penetration; this partly could explain the psychotropic properties of mitragynine reported in literature [22,23]. We previously reported a low content of mitragynine (31–444 µg/mL) in ketum decoctions often used by drug users to wean themselves off opiate withdrawal effects [24]. This may further explain the psychotropic properties of mitragynine owing to its intermediate lipophilicity which favours high BBB penetration. However, this warrants further detailed investigations using appropriate BBB studies. 

It is also important to note that mitragynine is a basic drug and likely to be charged in the intestinal lumen; as such to understand its lipophilic properties under various pH influence, logD values at pH 4, 7 and 9 were further determined. Despite being highly ionized in acidic solution, the drug has a logD value of 0.78 at buffer pH 4 thus indicating its preference to be distributed into lipid layer. The poor water solubility of mitragynine could be partly attributed to its lipophilicity properties. With regards to stability, mitragynine is stable at pH 7 and 9 for a period of 24 h. However, at pH 4 it appears to degrade over time and there is a high possibility the drug is degradable in gastric juice. In our previous study, we demonstrated the low absolute oral bioavailability (≈3%) of mitragynine in rats [11]. In the highly acidic environment of the stomach, drugs that have weak base mainly exist in their ionic form and are not readily absorbed through the cell membranes of the GI tract [25,26]. Mitragynine being a basic drug is highly solubilised and ionized in the stomach, thus making it less bioavailable. In addition, poor oral bioavailability was also likely to be caused by its degradation in the highly acidic gastric juice.

Other investigators have also reported the pharmacokinetic properties of mitragynine in rats after oral administration but the pharmacokinetic parameters varied largely among these studies. The reported value of C_max_ and half-life varied from 0.4–2.3 µg/mL and 3.8–9.4 h respectively after an oral dose ranging from 20–50 mg/kg [11,12,13,14]. These investigators used 1% acetic acid (pH 4.7), propylene glycol or 1% cremophor in saline respectively as a vehicle to facilitate the solubility of mitragynine for either oral or intravenous (i.v.) drug administration. In a separate study, Macko *et al* employed mitragynine salt solution for toxicity testing and reported no adverse reactions up to 920 mg/kg (mice) and 806 mg/kg (rats); a dose far higher than the doses reported by other investigators [9,10,13,15]. This is probably attributed to the fact that in salt solution mitragynine is highly in an ionized state and not readily absorbed from stomach. Further to this, the drug is not protected from degradation by the highly acidic gastric juice. This probably explains the non- manifestation of toxicity even at higher doses tested. Similar variability in analgesic and toxicity studies were also encountered when investigators employed various vehicles such as 4% acacia gum, tween 20% and 1% acetic acid adjusted to pH 4.7 for either oral or intraperitoneal (i.p.) drug administration [8,9,10,15]; this was done without proper understanding of its physicochemical properties and rationally this would have an effect on the outcome of their pharmacological studies [8,12,14]. The use of co-solvents or surfactants might enhance the *in vitro* solubility for the poorly soluble drug but its solubility *in vivo* might be totally different. With mitragynine, being a poor water soluble alkaloid, there is a possibility of this compound getting precipitated in the gut when given at large doses. Taking all possibilities into account, this may give an explanation for the wide-ranged disparity in the reported MG preclinical studies. 

Besides the above reported pharmacological studies, the addictive potential of mitragynine has been recently reported in humans and rodents [5,6]. Yusof *et al.* reported in their study on the addictive potential of mitragynine in rats where such effects were observed at high (30 mg/kg) and moderate (10 mg/kg) doses, but were absent at mild (5 mg/kg) and low (1 mg/kg) doses [6]. Their study involved intraperitoneal injection of mitragynine dissolved in the surfactant Tween 80. To the best of our knowledge, the first human study on the effects of mitragynine was performed by Grewal, but nothing on addictive properties was reported [27]. In Grewal’s study, the volunteers received mitragynine acetate (50 mg in 4 occasions) or ketum powdered leaves (0.65–1.3 g) with 20 mL of distilled water, in which case the solubility of the drug could be a limiting factor to oral absorption. Of late, Singh *et al.* in his psychosocial study reported that ketum users developed addiction only after prolonged, frequent and chronic consumption of high doses of ketum drinks prepared as leaves concoction, thus not mitragynine *per se* [5]. Interestingly, in a receptor interaction study using HEK 293 cells, ketum powder demonstrated a 350-fold lower affinity for the µ opiate receptor when compared to morphine [28]. This partly could explain the fact that regular consumption of high dose ketum drinks is required to observe abuse liability in humans. On the other hand, there is also a report on the antinociceptive effect (*i.e.*, mediated via opiate receptor) of mitragynine being less potent than the *M. speciosa* crude extract [29]. Presently, we cannot exclude that existing discrepancies between and within studies may be due, but not limited to, the route or the mode of administration. This further highlights the necessity to establish the physicochemical characteristics of mitragynine in order to prepare a suitable pharmaceutical formulation which would provide adequate absorption for investigations in future.

With reference to mitragynine stability, the drug was found to be stable in SIF compared to SGF over time of incubation (37 °C). A deviation above 5% of the original drug content, such as measured in the manner presented in this study is suggestive of drug instability in the GI tract. Mitragynine was not stable in SGF (pH ≈ 1.2) since drug loss was observed (>20%) at 20 min onwards after the onset of the incubation period. A similar drug loss was reported for mitragynine in SGF (26%) [30]. In the present study mitragynine degradation was further evident as large variation in drug release (>50%) was apparent in SGF dissolution profile with a total drug release up to 2 h which was only 80%. This is most likely due to the fact that the drug started to breakdown immediately upon release from its capsule after the onset of dissolution. 

However, mitragynine was stable up to 3 h throughout the incubation period in SIF (pH 6.8) with <5% deviation. Based upon these results, mitragynine is considered stable in SIF (3 h). Since the exposure of drug substances at 37 degrees Celsius to SIF (3 h) mimics the *in vivo* drug contact with these fluids, it was concluded that mitragynine could resist the pH and enzymatic conditions of the intestine fluid. However in SIF, mitragynine dissolution over time was prolonged and incomplete. After onset of the dissolution, only 22% of drug was released in 2 h. The drug in its pure basic form though stable in SIF, demonstrated a very poor aqueous solubility. SIF with a pH of 6.8, along with its chemical constituents and presence of enzyme certainly had an effect on the drug solubility. This was reflected in the low solubility of the drug in SIF when compared to its high solubility in acidic media. Though the amount of drug substance that goes into solution in SGF is very much higher than SIF, mitragynine in its ionized form is unlikely to be absorbed from the stomach. 

It is likely that mitragynine is better absorbed in the basic environment of the intestine owing to its lipophilic nature and the fact it predominantly exists in non-ionized form. However it is an acid labile drug and requires protection from acidic gastric juice when the drug is administered orally. In recognition of these physicochemical properties and to further improve its solubility, stability and to achieve uniformity in oral absorption, incorporation of mitragynine into a lipid carrier is essential. In the present work, physicochemical studies indicated that mitragynine is both hydrophobic and lipophilic in nature. Though it is poorly water soluble (<100 µg/mL) the drug showed some reasonable degree of solubility in lipid (logP: 1.70). This information is important as the formulation options available for hydrophobic or lipophilic compounds also differ considerably [31]. Perhaps employing techniques such as solid dispersion and by incorporating mitragynine into lipid carriers the drug solubility in aqueous media could be improved, since this vehicle acts by self-emulsifying the drug particles into fine divided state and simultaneously protects it from acid degrading. However, this warrants a detailed investigation using various lipid base carriers.

## 3. Experimental Section

### 3.1. Experiments

#### 3.1.1. Chemicals and Reagents 

Buffers, chemicals and reagents used were of analytical/chromatography grade and commercially sourced. High quality pure water was prepared using Millipore purification system (Model Elix SA 67120, Millipore, Molsheim, France). Glacial acetic acid and sodium hydroxide were procured from QREC (Chonburi, Thailand) and R&M Marketing (Essex, UK) respectively. Tris buffer and sodium carbonate were obtained from Acros Organic (Morris, NJ, USA) and Riedel-de Haen (Seelze, Germany) respectively. Sodium chloride was purchased from PROLABO (Paris, France). HPLC-grade acetonitrile was purchased from Fisher Scientific (Fair Lawn, UK) and formic acid and hydrochloric acid were purchased from Merck (Darmstad, Germany).

#### 3.1.2. Extraction and Isolation of Mitragynine

Leaves of *M. speciosa* were collected from Perlis, Malaysia and were identified by a botanist in the School of Biological Sciences, Universiti Sains Malaysia (Penang, Malaysia). A voucher specimen (No 11074) was deposited at the herbarium of the School of Biological Sciences, USM for further reference. Mitragynine was isolated following procedures as described previously [32].

#### 3.1.3. Spectrophotometry

The instruments employed were UV spectrophotometer 8453 UV-Vis Diode Array (Agilent, Santa Clara, CA, USA), and Multiskan* GO Microplate Spectrophotometer (Thermo Scientific, Waltham, MA, USA). Data were captured using the Thermo Scientific SkanIt Software (Thermo Scientific, Version 3.2). Buffer Maker Software (version 1.0.1.55, BPP Marcin Borkowski, Marki, Poland,) was used for preparation of buffers with constant ionic strength for pK_a_ determination. The pH-meter employed throughout the study was a PH 211 Hanna (Padova, Italy) equipped with a combined glass electrode.

#### 3.1.4. HPLC

The HPLC system consisted of a Jasco delivery pump (Tokyo, Japan) equipped with autosampler connected to a Waters 2487 detector (Milford, MA, USA) coupled to Jasco Chromnav software (Version 2.0, Tokyo, Japan). Chromatographic separation was performed on a C_8_ Agilent Eclipse plus (4.6 mm i.d. × 250 mm, 5-μm particle size; Apple Valley, MN, USA). The HPLC-UV method was adapted from our previous study [24] and revalidated for the present HPLC system. The method was employed to quantify mitragynine in partition coefficient, stability, dissolution and solubility studies. 

#### 3.1.5. pK_a_

pK_a_ determination was carried out using conventional UV and microplate spectrophotometer methods. Acetic acid, tris base and sodium carbonate were used to prepare stable buffers in the pH range of 3 to 12. The buffers were prepared to give a final buffer molarity of 0.01 M. Either NaOH or HCl were used for pH adjustment of the various buffers, whose ionic strength was kept constant at 0.02 by addition of NaCl.

#### 3.1.6. Conventional UV Method

Five mL aliquots of buffer of various pH were transferred into separate tubes. To each tube, mitragynine stock solution in acetonitrile (4 mg/mL) was added to give a final drug concentration of 20 µg/mL. The percentage of organic content in final buffer preparation was kept below 1%. The prepared solutions were immediately scanned in the range of 200–400 nm.

#### 3.1.7. Microplate Method 

Stock solution of mitragynine at 0.5 mg/mL was prepared in acetonitrile. Ten microliters of the stock solution was transferred into individual test tubes and diluted with 1.0 mL buffer of various pH to give a final concentration of 5 μg/mL. For absorbance spectrum analysis, 250 μL of the various pH buffer solutions spiked with drug were placed in a 96-well quartz UV-transparent microplate and immediately scanned in the range of 200–400 nm at a scanning rate of 200 nm/s in triplicates. The pK_a_ was calculated with equation pK_a_ = pH + log (d − dm/di − d), for basic compound, where di is the absorbance of the ionised species, d is the absorbance of the solution tested and dm is the absorbance of the neutral species [17]. 

#### 3.1.8. Solubility and Stability

For solubility studies, an excess of mitragynine was added to 1.0 mL each of distilled water or buffers at pH 4, 7 and 9. The test tubes were placed in a water bath shaker at 37 degrees Celsius at 100 cycles/min for 48 h until equilibrium. The samples were centrifuged at 3000 rpm for 5 min and supernatant was taken for quantification of mitragynine by HPLC-UV. For stability studies, analogous buffers were used, but they were spiked with mitragynine in acetonitrile to give a concentration of 20 µg/mL (organic content was kept below 1%). A 0.1 mL aliquot was collected from each buffer system at intervals of 0, 0.5, 6.0 and 24.0 h for determination of mitragynine. Mitragynine concentrations were expressed as percentage recovered. All experiments were performed in triplicates.

#### 3.1.9. Partition Coefficient 

Lipophilicity of mitragynine was determined by n-octanol/water partition coefficient (P) using the method described by Low *et al.* [33]. The distribution coefficient (log D) was determined in a similar manner to logP but instead of using water, buffers with a specific pH (4, 7, and 9) were used.

### 3.2. Mitragynine Stability in Simulated Gastrointestinal Fluids 

In this experiment the stability of pure mitragynine standard in simulated gastric fluid (SGF) and simulated intestinal fluid (SIF) was determined. The SGF was prepared according to USP specifications. For SGF, 2.0 g sodium chloride and 3.2 g pepsin (from porcine stomach mucosa) were dissolved in 7.0 mL hydrochloric acid. Then water was added to make up 1000 mL solution. The SGF pH was around 1.2. For the SIF, 6.8 g monobasic potassium phosphate was dissolved in 250 mL water. To this, 77 mL 0.2 N sodium hydroxide 500 mL water and 10.0 g pancreatin (from porcine pancreas) was added and mixed. The SIF solution was adjusted to pH 6.8 with either 0.2 N sodium hydroxide or 0.2 N hydrochloric acid and further diluted with distilled water to 1000 mL. The stability of mitragynine in SIF and SGF experiments was carried out in 10 mL volumetric flasks respectively. One hundred microliters (100 µL) of the drug solution (2 mg/mL) was spiked into flasks containing preheated SGF and SIF (10 mL) at 37 °C respectively (organic content was kept below 1%). The flask was placed in a 37 °C shaking water bath. For SGF study 400 µL of samples were drawn at 0 and thereafter at 10, 20, 30, 40, 50, 60, 70, 80 and 90 min after onset of the experiment. The samples were collected into micro-centrifuge tubes and centrifuged at 9000 rpm for 10 min and the supernatants were transferred into test tubes. After that, 150 uL of the supernatant was diluted with acetonitrile (1:1) and injected into HPLC. The similar procedure was carried out for SIF but the sampling time was extended to 3 h (e.g., 120, 180 min). Both SGF and SIF samples were analyzed in triplicates. The relative difference (RD) between the amount of mitragynine added at the beginning and that determined at the different intervals of the incubation period was calculated as follows:

RD = (C_a_ − C_b_)/C_a_ × 100%

where C_a_ is the amount of drug found at the zero time point and C_b_ is the amount found at the end of incubation. It gives a measure of the extent of any degradation of the drug in the presence of the gastrointestinal fluids.

### 3.3. In Vitro Release Test of Mitragynine from Capsules Filled with Mitragynine Pure Standard

Hard shell gelatin capsules filled with mitragynine pure standard were used in the *in vitro* dissolution study. In order to characterize the release of mitragynine, drug release profile was generated, in which mitragynine release values were determined as a function of time. The US pharmacopoeia I (basket) method was used for the *in vitro* dissolution study. SGF (pH 1.2) and SIF (pH 6.8) were prepared without enzymes [34]. These media were used for the dissolution study, respectively. The capsule filled with mitragyine was introduced into 500 mL of dissolution medium. The capsule was agitated at 50 rpm at 37 °C. An aliquot (1 mL) of sample was withdrawn at predetermined time intervals of 5, 10, 15, 30, 40, 60, 90 and 120 min after onset of the dissolution. The samples were filtered through 0.45 um PTFE syringe filters, diluted with ACN (1:1) and the drug concentration was determined by the validated RP-HPLC-UV method. Samples were analyzed along with QC samples. 

## 4. Conclusions 

In the present work, our findings indicated that mitragynine is a weak base, with intermediate lipophilicity, poorly soluble in water and basic media, highly soluble in acidic media, but acid degradable. It is found to be unstable in SGF and stable in SIF but poorly goes into solution in SIF.These findings will assist investigators towards designing a more appropriate formulation for preclinical studies that mitigate the problems associated with the poor absorption and stability of mitragynine. It is imperative to establish a more realistic preclinical assessment of the compound before one can make a correct inference from animal studies for human safety risk prediction. 
